# Towards time-resolved MicroED grid preparation using mix-and-inject gas dynamic virtual nozzles

**DOI:** 10.1107/S2052252526005129

**Published:** 2026-06-23

**Authors:** Jacob A. Summers, Niko W. Vlahakis, Kara A. Zielinski, Sarah Uttormark, Scout Fronhofer, Cole Dolamore, Mark A. Wilson, Lois Pollack, Jose A. Rodriguez, Peter D. Dahlberg, Soichi Wakatsuki

**Affiliations:** a Department of Structural Biology Stanford University School of Medicine Stanford CA94305 USA; b Department of Chemistry and Biochemistry University of California Los Angeles Los Angeles CA90095 USA; c Department of Energy Institute for Genomics and Proteomics University of California Los Angeles Los Angeles CA90095 USA; d Science and Technology Center on Real-Time Functional Imaging, NSF Science and Technology Center University of California Los Angeles Los Angeles CA90095 USA; e School of Applied and Engineering Physics Cornell University Ithaca NY14853 USA; f Department of Biochemistry University of Nebraska-Lincoln Lincoln NE68588 USA; g Stanford Synchrotron Radiation Lightsource SLAC National Accelerator Laboratory Menlo Park CA94025 USA; European Molecular Biology Laboratory, France

**Keywords:** MicroED, time-resolved studies, gas dynamic virtual nozzle, proteinase K

## Abstract

Recent progress in gas dynamic virtual nozzle (GDVN) technologies in combination with high-brilliance synchrotron and X-ray free-electron lasers (XFELs) has allowed the visualization of protein dynamics *in crystallo* by mixing macromolecular protein crystals with a substrate using tunable mixing times on the order of milliseconds to seconds prior to serial X-ray diffraction data collection. This has become the method of choice for high-resolution structure determination of intermediate states. However, such experiments require large counts of crystals of proper sizes for high-resolution data collection, and premium beam times for screening efforts. Cryogenic microcrystal electron diffraction (MicroED) represents a complementary technique that may be a more accessible avenue for time-resolved nanocrystallography compared with serial X-ray diffraction experiments. MicroED can produce full diffraction datasets from just a few submicrometre-thick crystals, and the approach is more readily accessible, requiring standard cryogenic transmission electron microscopy (TEM) equipment available at many universities and institutes. Cryogenic MicroED, like other forms of cryo-EM, begins with rapidly freezing biological material on electron microscopy grids. In the case of MicroED, micro- to nano-crystals (<500 nm thick) are deposited onto electron microscopy grids and plunge-frozen for subsequent electron diffraction data collection. Here, we have incorporated GDVN technology developed originally for XFEL experiments into the freezing process as a first step towards time-resolved studies. We describe the limited deposition efficiency of the model MicroED protein proteinase K on TEM grids using GDVNs, preceding sample vitrification and successful MicroED data collection. We discuss both the initial results from such experiments and the methodological challenges in developing this approach into a reliable workflow for millisecond-to-second time-resolved structural studies of macromolecules. Our results promise a strategy to deposit crystals on grids using GDVNs and determine high-resolution structures by MicroED, constituting a first step towards development of time-resolved MicroED experiments.

## Abstract 2

This article establishes the feasibility of time-resolved microcrystal electron diffraction by using a microfluidic mixing device to deposit microcrystals onto electron microscopy grids during plunge-freezing. The resulting vitrified crystals were of sufficient quality to solve a model protein structure.

## 1. Introduction

Protein crystallography is a crucial tool in developing our understanding of the relationship between protein structure and function. Synchrotron X-ray diffraction (S-XRD) of biomolecules is the most commonly used method for efficient protein structural characterization and is readily applied to ligand-bound protein complexes for application in drug-discovery efforts. However, S-XRD presents challenges when a protein of interest does not easily crystallize, or if the achievable crystals are small. Moreover, it typically can only deliver static structures which poorly represent the critical temporal context of what are known to be inherently dynamic systems. Therefore, dynamics are typically inferred with the assistance of computational methods, such as molecular dynamics, or lower-resolution experimental methods such as single-molecule fluorescence resonance energy transfer (smFRET; Roy *et al.*, 2008 ▸). However, reacting biomolecules with substrates *in crystallo* historically provides an experimentally verified context to inferred dynamics, as has been demonstrated for years (Hajdu *et al.*, 1987 ▸; Moffat, 1998 ▸; Schotte *et al.*, 2004 ▸). Furthermore, recent progress in microfluidic technologies coupled with the development of X-ray free-electron lasers (XFELs) has allowed more control over the visualization of protein dynamics *in crystallo* by mixing macromolecule crystals with a substrate for tunable mixing times on the order of milliseconds to hundreds of milliseconds prior to XFEL diffraction (Calvey *et al.*, 2019 ▸; Caramello & Royant, 2024 ▸).

Time-resolved XFEL (TR-XFEL) experiments allow high-resolution structures of intermediates to be resolved by coupling the brilliance of an XFEL source with rapid substrate diffusion into microcrystals for subsequent enzymatic interaction. By using microcrystals (typically <50 µm thick) with open solvent channels accessible to the substrate-binding sites, diffusion times of substrates into crystals can occur on the order of milliseconds to microseconds, depending also on the substrate size and diffusion coefficient, and the solvent-channel width; furthermore, fast mixing times can enable the use of a broader range of solvents which may otherwise damage crystal integrity (Schmidt, 2013 ▸). Due to the small X-ray beam sizes and brilliance of XFELs, much smaller crystals can be used for diffraction experiments compared with S-XRD; however, such experiments require competitive XFEL beamtimes and a high number of microcrystals, creating significant limitations for routine experiments. Further, prior to a TR-XFEL experiment, mixing and sample conditions must first be optimized through standard XFEL experiments. Thus, in the best case, these experiments can take months to years to conduct, and this neglects the downstream data analysis, which is computationally challenging and storage-intensive and in general is nontrivial to carry out. However, this is the best-case scenario, and for many systems where crystals are small, and sample quantity is limited, this route can be altogether intractable.

Microcrystal electron diffraction (MicroED) offers an attractive alternative addressing the challenges that S-XRD and TR-XFEL present, although it presents its own challenges and limitations. The application of MicroED is still very much in early growth stages when compared with S-XRD and XFEL and has yet to be widely adopted for structure and dynamics studies. Despite the infancy of MicroED, transmission electron microscopes (TEMs) have been used to assess microcrystal quality for SFX experiments (Stevenson *et al.*, 2014 ▸).

MicroED involves rapidly freezing samples of micro- to nano-crystals <500 nm thick by plunge-freezing (Dobro *et al.*, 2010 ▸; Shi *et al.*, 2013 ▸) onto electron microscopy grids for continuous rotation diffraction (Nannenga *et al.*, 2014 ▸) using TEM. Crystals must be thin enough for electron transmission, which due to the high scattering cross-section of electrons and matter limits the mean free path of electrons to roughly 300 nm (Saha *et al.*, 2022 ▸). This large cross-section results in a high likelihood of multiple elastic and inelastic scattering events per electron from thick samples, which can produce inaccuracies in integrated intensities or obscure Bragg reflections, respectively (Martynowycz *et al.*, 2022 ▸). Further, the accessible tilt range of the TEM rotation stage is limited to a maximum of ∼140°. For crystals with low-symmetry space groups, data from multiple crystals must be merged to avoid ‘missing wedges’ in reciprocal space. Preferential orientation of crystals on the EM grid can further lead to the absence of data in certain volume wedges in reciprocal space despite exhaustive merging, resulting in lower data completeness with resolution anisotropy and in density maps with less clarity in certain directions.

Despite these limitations, MicroED remains a powerful and growing method for structural biology, capable of resolving protein structures to resolutions of 2–3 Å or finer if well diffracting microcrystals can be achieved (Clabbers *et al.*, 2025 ▸). MicroED also has particular advantages for time-resolved studies. Namely, the thin crystals most ideal for MicroED allow rapid diffusion of potential ligands (Clabbers & Xu, 2020 ▸; Mu *et al.*, 2021 ▸; Vlahakis *et al.*, 2025 ▸). If larger crystals (micrometres in thickness) are required, recent advances have integrated cryogenic focused ion beam (FIB) milling (Martynowycz & Gonen, 2021 ▸) to ablate unwanted regions of microcrystals and obtain MicroED from the resulting thin lamellae; however, even these large crystals are smaller than those typically used in S-XRD experiments. Importantly, MicroED consumes far less sample than that needed for serial XRD. Typical sample needs are measured in single microlitres and are at similar concentrations to XFEL- and S-XRD-based approaches that require millilitres. Further, while stage rotation is limited, nearly complete structures can be obtained from just a few crystals (Yonekura *et al.*, 2015 ▸) or even a single crystal if it has high symmetry (Clabbers *et al.*, 2017 ▸). This also makes it possible to identify multiple crystal forms within the same crystallization preparation. Lastly, compared with XFEL- and S-XRD-based approaches, MicroED relies on significantly more accessible equipment, primarily requiring a plunge-freezing apparatus and a cryogenic TEM. However, despite the potential of MicroED to bridge the gap between XFEL and serial synchrotron crystallography methods, there has not previously existed a robust method to perform time-resolved experiments by MicroED.

One reason for the absence of a developed field of time-resolved MicroED is the lack of robust grid-preparation methods. In the field of time-resolved single-particle cryo-EM, many methods have been developed, such as the Spotiton device, which uses a polydimethylsiloxane-based mixer and microsprayer to spray mixed samples onto a TEM grid (Dandey *et al.*, 2020 ▸). Another similar method uses a microfluidic mixing channel coupled with a piezoelectric sprayer to deposit mixed samples onto grids with subsecond precision (Feng *et al.*, 2017 ▸). However, these methods are designed to mix two fully soluble components together and their ability to mix crystals is untested.

Recently, we have demonstrated the use of mix-and-inject gas dynamic virtual nozzles (GDVNs) for TEM grid preparation for time-resolved cryogenic electron tomography (Yoniles *et al.*, 2024 ▸). In these experiments, we were able to rapidly freeze whole bacterial cells that had been mixed with a low-pH buffer for controlled times prior to freezing. Subsequent cryogenic electron tomography showed time-dependent structural changes of surface-layer 2D protein lattices, outer membranes and cytosolic macromolecular complexes in the whole cells. Reaction times for this previous work covered 250–1500 ms, with ∼10–100 ms precision, where the time range and precision were largely determined by the microfluidic mixer chosen. The success of this mix-and-spray technology encouraged us to extend the approach to time-resolved mix-and-spray MicroED as both a standalone method and as a complementary method for mix-and-inject S-XRD and XFEL studies. As substrate diffusion in nanocrystals optimal for MicroED occurs on timescales faster than those currently achievable with traditional MicroED grid-preparation methods, we used a GDVN-assisted deposition approach to reduce the grid-preparation time towards timescales amenable to time-resolved MicroED studies. Here, we describe the first attempts of MicroED using mixing GDVN jets for sample preparation and discuss the results and future technical challenges in developing this into a reliable workflow for fast time-resolved structural dynamics studies of macromolecules.

## 2. Methods

### 2.1. Crystallization

Slurries of proteinase K (ProK) crystals were prepared by the sitting-drop method. Proteinase K from *Tritirachium album* was commercially acquired from GoldBio, resuspended in 50 m*M* HEPES pH 7.0 at 50 mg ml^−1^ and mixed at a 1:1 volume ratio with crystallization solution (1.2 *M* ammonium sulfate, 0.1 *M* Tris–HCl pH 8.0) in large-well sitting-drop plates (Hampton Research, catalogue No. HR3-308). Needle-shaped crystals were allowed to grow for 24–48 h before being crushed by repeated pipetting of the well solution and pooled together to produce a concentrated crystal slurry.

*Candida albicans* heat-shock protein 31 (Hsp31; also called Glx3) was purified as described previously (Hasim *et al.*, 2014 ▸). A new crystal form was prepared in 1.2 *M* citrate at pH 6.5 by sitting-drop vapor equilibration and allowed to grow for approximately one week at room temperature before being harvested. These crystals grew as thin (≤1 µm) plates that diffracted X-rays poorly, making them compelling candidates for MicroED.

### 2.2. Grid preparation

Grids were plasma-etched for 60 s per side at 15 mA using a PELCO easiGlow system (Ted Pella, catalogue No. 91000). 400-mesh lacey carbon grids with 2 nm continuous carbon coating (catalogue No. LC400-Cu-CC) were found to present the thinnest ice while maintaining a majority of intact squares during preliminary testing (data not shown), so they were used for all subsequent data-collection steps using spray-frozen samples. A minimally modified Gatan CP3 was used for plunge-freezing grids through the path of mixed droplets and microcrystal slurry into temperature-controlled liquid ethane (Yoniles *et al.*, 2024 ▸). For comparison, grids were also prepared using traditional MicroED methods. 300-mesh copper Quantifoil grids (catalogue No. Q350CR1.3) were plasma-etched following the previously described protocol. For proteinase K, crystals were diluted 1:10 in 20 m*M* Tris buffer pH 8.0, and 2 µl crystal slurry was applied to the grids. Excess liquid was blotted from the grids using a Vitrobot Mark IV (Thermo Fisher Scientific). Hsp31 crystals were applied directly to grids without dilution, and excess liquid was blotted by manually contacting the back of the grid within a humidified Vitrobot chamber. Although the high concentration of sodium citrate in the Hsp31 crystallization condition risked the formation of salt crystals on TEM grids during either preparation scheme, it did not appear to prohibit proper vitrification of the sample with either method.

### 2.3. Mixer fabrication and operation for MicroED grid preparation

Microfluidic mixers were coupled to GDVNs as in our previous work (Calvey *et al.*, 2016 ▸, 2019 ▸). Capillaries (75–100 µm inner diameter, 200 µm outer diameter; Polymicro Technologies, Phoenix, Arizona, USA) were polished and beveled, and Kapton centering spacers were placed on the tip. The delay-line capillary was glued inside a piece of glass, and the supply-line capillary was secured upstream of the delay line with standard fittings. The GDVN nozzle was formed by flame polishing and the mixer was inserted inside the nozzle.

For sample deposition onto grids, microcrystals were first filtered through a 25 µm mesh and then loaded into a reservoir (Neptune Fluid Flow Systems, Knoxville, Tennessee, USA), while stabilizing buffer was loaded into a separate reservoir to serve as sheath flow. Both reservoirs were connected to HPLC pumps (LC-20AD from Shimadzu Scientific Instruments) to drive liquid delivery. In GDVNs, gas is used to create a free-standing liquid jet, but here nitrogen gas was used to generate a spray with a gas flow rate of 160–215 mg min^−1^ rather than the typical 30–40 mg min^−1^.

The nozzle was mounted on a positioning stage for precise alignment to the grid, with a shutter in front to avoid pre-wetting the grid. Tweezers holding the grid were placed onto the plunge-freezer arm. After removing the shutter, plunging was triggered to move the grid through the liquid spray, collecting the sample before the grid was rapidly vitrified in a cup of liquid ethane. Room humidity was not maintained during the freezing process.

### 2.4. TEM imaging of samples and MicroED data collection

Vitrified samples were evaluated using various microscopes and detectors. For preliminary evaluation of ice distribution and crystal deposition efficiency onto grids, a 300 kV electron microscope (Titan Krios, Thermo Fisher Scientific) equipped with a direct electron detector (Gatan K3) and energy filter was used, and imaging was performed using *SerialEM* (Mastronarde, 2005 ▸). To further evaluate the ice distribution and diffraction quality of embedded crystals, a 200 kV electron microscope (Glacios 2, Thermo Fisher Scientific) equipped with a diffraction-optimized detector (Ceta D, Thermo Fisher Scientific) was used, and diffraction tilt series were collected in *EPU-D* (Thermo Fisher Scientific). Grids on which crystals were observed, embedded in sufficiently thin ice, were moved forward for full data collection using a 300 kV electron microscope with diffraction-optimized detector (Titan Krios and CetaD, Thermo Fisher Scientific). Diffraction data were collected and saved in SMV format using *EPU-D*. Data were acquired in nanoprobe mode with an illuminated area of approximately 4 µm, where each crystal positioned eucentrically within the illuminated area was rotated to collect a series of 1° rotation images at a rate of either 0.1 or 0.2° s^−1^ while the detector acquired 10 or 5 s integrated exposures, respectively. Such continuous rotation datasets were acquired from many crystals, enabling scaling and merging of partial datasets. For the structure of proteinase K microcrystals prepared using ashless blotting conditions, the same scheme as above was performed, instead using a 200 kV electron microscope (Glacios with CetaD detector, Thermo Fisher Scientific) and acquiring data from crystals using microprobe mode with a selected area aperture projecting a circular area of approximately 2.5 µm diameter on the specimen.

### 2.5. Data processing

MicroED data in SMV format were processed as follows: reflections were indexed and integrated using *XDS* (Kabsch, 2010 ▸) and scaled and merged between partial datasets using *XSCALE*. Phases were determined by molecular replacement with a previously determined structure of proteinase K from X-ray diffraction (PDB entry 2id8) using *Phaser* and refined in *REFMAC*.

## 3. Results

### 3.1. Determining optimal microcrystal deposition parameters for mix-and-spray MicroED

**Figure 1:**
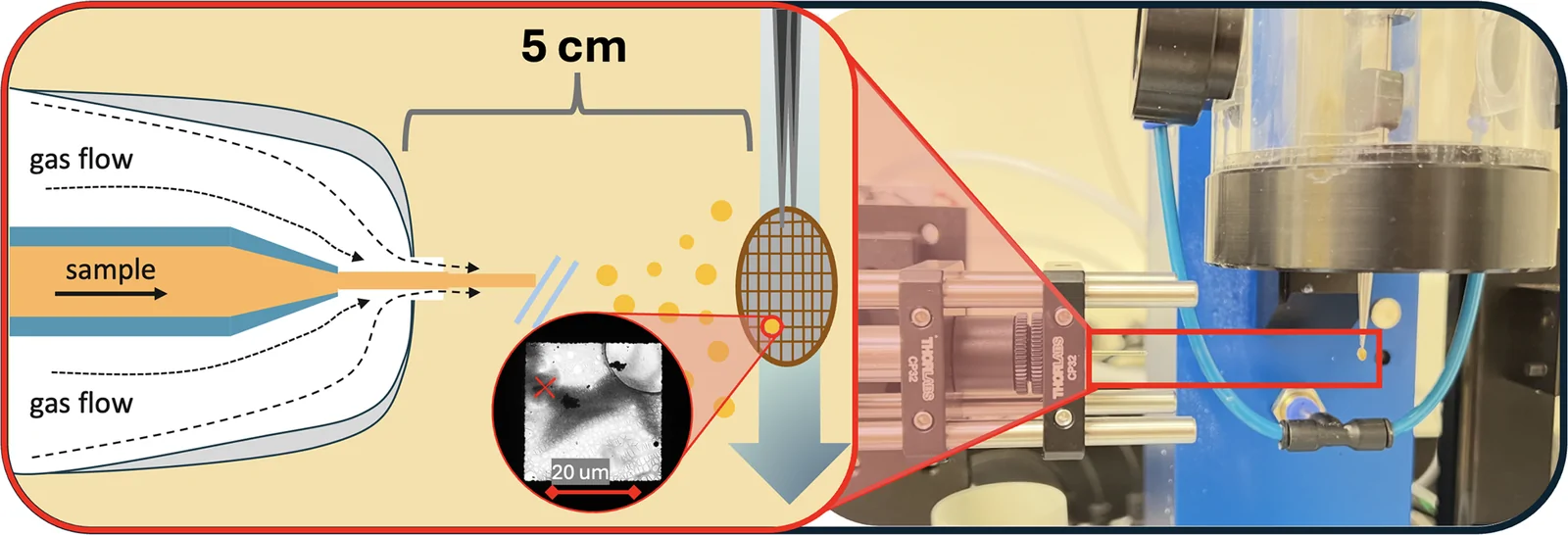
Diagram of mix-and-spray setup for microcrystal deposition onto TEM grids. Microcrystals are propelled through the nozzle (*a*) via an HPLC pump and droplets are formed and focused using nitrogen gas. Droplets are propelled across a 5 cm space onto grids driven by gravity into liquid ethane. Adjustment of both liquid flow rate and gas speed allows optimization of droplet size and distribution onto the grid. (*b*) Physical apparatus highlighting the grid, tweezers, isolated chamber and nozzle. Tweezers are shown in a mid-plunge position after exiting the shuttered chamber above.

We assembled a spray-freezing apparatus based on a previously demonstrated mix-and-spray device used for whole-cell cryo-ET grid preparation (Yoniles *et al.*, 2024 ▸). We optimized our setup to spray microcrystals by increasing the diameter of the inner sample capillaries to carry our crystals without clogging the jets. To prevent crystal settling, we introduced a crystal anti-settler to keep the crystals suspended within the reservoir as we ran the system. Once a stable spray was achieved at a given sample and gas flow rate, we kept the sample running in between loading new grids into the tweezers in order to maintain stable sample delivery and prevent clogging. Custom shutters were prepared from sponges and installed in front of the nozzle in order to absorb samples coming from the jet while loading the tweezers onto the CP3. Before the deposition, the loaded tweezers were located in an isolated chamber above the spray, so no pre-deposition of sample is possible between removing the sponge shutter and plunging the grid into liquid ethane (Fig. 1 ▸).

Due to the high gas pressures and sample flow rates used for the mix-and-inject GDVN, we found that certain TEM grid types were too fragile for this application and some grid types maintained a preferential ice thickness and distribution for diffraction (Supplementary Fig. S1). We ultimately proceeded with grids of 400-mesh lacey carbon with a 2 nm continuous carbon layer for sample preparation and data collection. To ensure crystals were not damaged due to high gas flow rates or spraying, we collected crystals in an Eppendorf tube and then examined them under a stereoscope. There were no signs of visible damage and the crystals looked intact and were the same size and shape as the crystals before spraying. Additionally, the crystals captured on the grid also matched the size and shape.

**Figure 2:**
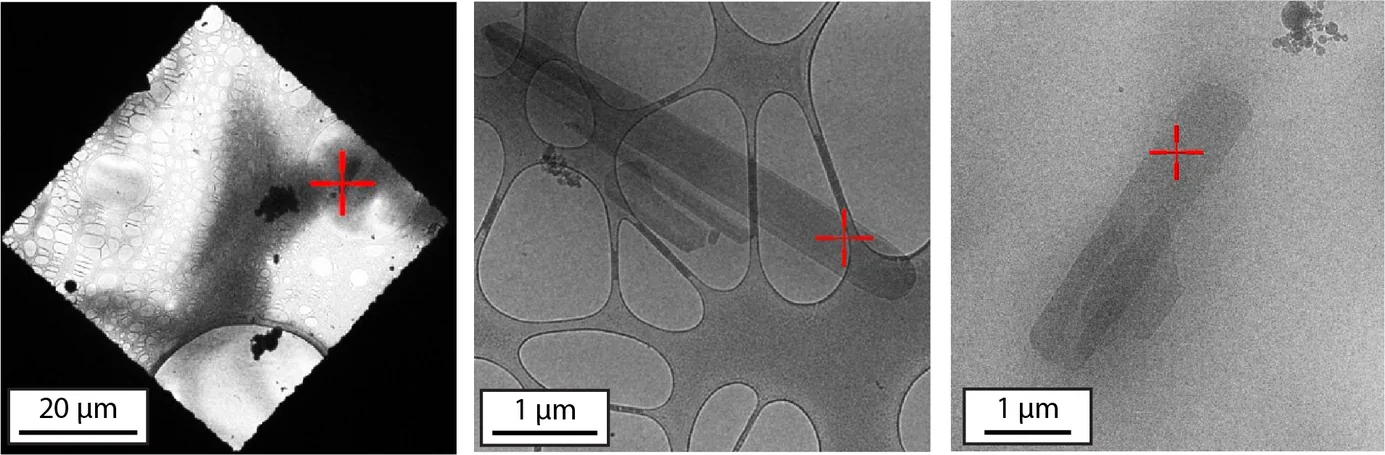
Typical examples of proteinase K microcrystals sprayed onto lacey carbon grids. Proteinase K microcrystals which were sprayed onto 400-mesh lacey carbon TEM grids were screened for ice thickness and diffraction quality prior to collection (the red cross-hair indicates the crystal).

MicroED from spray-frozen grids was initially frustrated by a low deposition rate of crystals, with most grids containing no crystals. However, through optimization of sample and gas flow rates, the crystal deposition efficiency was improved. A key variable controlling the amount of deposited material is the speed of the grid while it is transiting through the aerosol. If the grid is moving quickly, less material will be incident on the grid because it moves through the aerosol region in a short amount of time. The plunger is gravity-driven and thus the velocity increases as the square root of the distance traveled along the plunging path. This allowed an improvement to the amount of deposited material by decreasing the vertical distance between the plunger’s released position and the aerosol region. This effectively reduced the speed of the tweezers through the aerosol region, allowing more droplets to be caught by the grid, and only increased the uncertainty in mixing times marginally. Through this optimization, we achieved about one quality diffracting crystal per grid (Fig. 2 ▸). This is still far below ideal and limited this work to model systems despite attempts with nonmodel microcrystals. See the supporting information for further information on attempts with nonmodel microcrystals. Thus, at present, low microcrystal deposition rates per grid remain an open technical challenge for time-resolved MicroED.

### 3.2. Spray-freezing MicroED for structure determination of proteinase K

Having established freezing conditions that resulted in crystal deposition, we next wanted to determine whether the mix-and-spray deposition process was detrimental to the microcrystal diffraction quality by comparing spray-frozen MicroED structures with structures from traditional plunge-freezing. We selected a needle-shaped microcrystal polymorph of *T. album* proteinase K, which routinely diffracts to finer resolution than 2.5 Å by MicroED, as our initial reliable model system for addressing these questions (Supplementary Table S1). Microcrystals formed in batch were mixed with crystallization solution using the mixing jet. We screened for ice quality and diffraction using a 200 kV electron microscope (Glacios, Thermo Fisher Scientific) with a diffraction-optimized detector (CetaD, Thermo Fisher Scientific) and identified a set of gas and liquid flow rates for more efficient crystal deposition onto grids.

**Figure 3:**
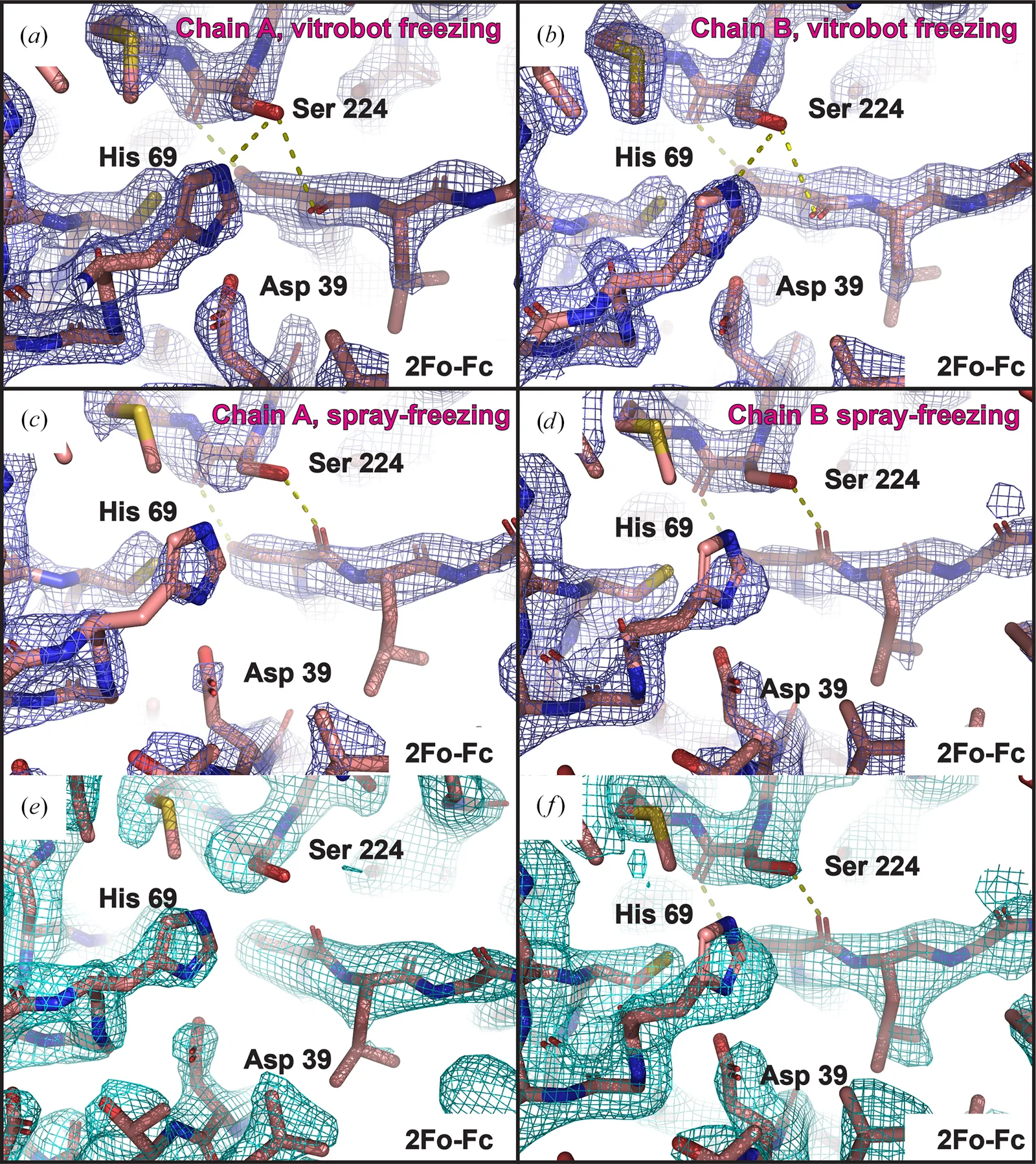
Structures of proteinase K determined by spray-freezing and traditional plunge-freezing methods. The apo-form structure of proteinase K crystals at 2.2 Å resolution prepared by standard blotting and plunge-freezing methods is superimposed with the 2*F*_o_ − *F*_c_ map at a 1.5σ level viewing the empty active site in chain *A* (*a*) and chain *B* (*b*) of the asymmetric unit. The active site is in good agreement with that of a 2.5 Å resolution structure determined exclusively from spray-frozen proteinase K crystals (*c*, *d*). (*e*) and (*f*) show the same views as (*c*) and (*d*), respectively, with the σ level of the 2*F*_o_ − *F*_c_ map reduced to 1.0.

After further optimization of ice quality by adjusting the gas and liquid flow rates and moving to a 300 kV electron microscope (Titan Krios, Thermo Fisher Scientific) to enhance the diffraction of crystals embedded in thicker ice, we then proceeded to solve a 2.50 Å resolution MicroED structure of spray-frozen proteinase K (Supplementary Table S2). The primary limitation of data acquisition from these crystals appeared to be data completeness, as the crystals suffered heavily from orientation bias on the TEM grid and overall deposition of crystals remained sparse. The final completeness we achieved was 83.4% from diffraction data merged from seven crystals, which was still sufficient to yield a structure in good agreement with a structure determined from the same crystals prepared by traditional freezing methods (Fig. 3 ▸, Table 1 ▸). Interestingly, the calcium ion often observed to occupy a site adjacent to Asp200 was observed in the control Vitrobot-frozen structure [Supplementary Figs. S2(*a*) and S2(*b*)], but was notably absent in the spray-frozen structure [Supplementary Figs. S2(*c*) and S2(*d*)]. We hypothesized that this calcium ion may have been introduced during the blotting process, as calcium might leech from the filter paper used for blotting during our 10 s blotting period. In order to test this hypothesis we repeated standard MicroED experiments using calcium-free ashless blotting paper (WHA1441055, Sigma–Aldrich). The resulting 2.20 Å resolution structure obtained again showed the absence of the calcium ion at this site [Supplementary Figs. S2(*e*) and S2(*f*) and Table S3], suggesting that calcium was indeed introduced during the blotting process. The missing wedge of the merged data in reciprocal space was also roughly consistent with that observed in the structure determined from traditionally Vitrobot-frozen crystals, suggesting that no additional orientation bias was introduced or substantially alleviated by the spray-freezing process (Table 1 ▸, Supplementary Fig. S3).

## 4. Discussion

This work is a proof-of-principle demonstration that lays the groundwork for future mix-and-spray MicroED. We demonstrated the ability to deposit microcrystals on grids and determine high-resolution structural information. The diffraction quality and resulting MicroED protein structures using our mix-and-spray sample-delivery system are comparable to those with the conventional blotting method.

The needle-shaped proteinase K crystal polymorph selected for initial experiments exhibited strong orientation bias on TEM grids, whether prepared by traditional plunge-freezing or spray-freezing methods. This produced a missing wedge of data encompassing the b* axis of reciprocal space for these crystals, and the low symmetry of the crystal form limited data completeness regardless of the sample-preparation method (Supplementary Fig. S3). Preferential orientation of low-symmetry crystals presents a particular challenge in the case of limited sample deposition, as was initially contended with in our spray-freezing experiments. Nevertheless, merged datasets of comparable completeness to what was readily achievable using traditional plunge-freezing were obtained from spray-frozen samples of proteinase K (Table 1 ▸). In addition, we attempted to spray-freeze a more challenging protein sample, Hsp31, and obtained comparable diffraction quality (see Supplementary Figs. S4 and S5).

This work was enabled by GDVN technology routinely used for TR-XFEL and synchrotron crystallography experiments. These mixers can easily be modified and incorporated into plunge-freezers for mix-and-spray MicroED sample preparation, and as this work demonstrates, data collection is now feasible with optimization. The cost and accessibility of TEM instruments for diffraction may provide an alternate route for screening diffusion-limited time-resolved crystallography projects and could grant another method for visualizing structural dynamics which may be lower risk when compared with time-resolved mix-and-inject XFEL experiments.

This work has also identified a key challenge with mix-and-spray MicroED sample preparation: achieving high sample density on the grid. We suspect that the extremely fast jetting (up to 100 km h^−1^) may lead to tumbling of the crystals, resulting in a large divergence of the spray, and may be part of the reason for the low efficiency in crystal deposition. This problem calls for optimization of the geometry, grid type, gas pressure and flow rates of mixing jets for crystal deposition. One challenge in optimizing sample density is the large parameter space that needs to be explored and the low throughput of screening EM grids. As part of this work, more than 100 grids were evaluated in order to identify more optimal parameters, requiring hundreds of hours of researcher effort and microscope usage, and while future experiments will undoubtedly be faster, easier methods of parameter screening need to be explored. We also anticipate further challenges as we expand to more complex systems, such as membrane proteins, as GDVNs have seen limited use with detergents and this may impact flow and the subsequent sample deposition. These additional sample-optimization requirements join those already present in MicroED for static, traditionally frozen samples, namely the narrowing down of crystallization conditions that produce the highest quality microcrystals while also posing as little of an obstacle to successful vitrification of a thin layer of ice about these crystals as possible. Although ice and crystal thickness seem to have limited our ability to achieve optimal diffraction from certain spray-deposited crystals we could identify on the grids, combining this technology with existing methodologies that address MicroED sample thickness, such as cryo-FIB milling (Martynowycz & Gonen, 2021 ▸), may further extend the ability to achieve optimal diffraction from time-resolved MicroED samples. While progress continues, this work is a significant milestone for the development of time-resolved MicroED by demonstrating that nanocrystals can be mixed within a microfluidic mixer and deposited onto TEM grids in a manner compatible with millisecond time-scale mix-and-spray crystallography methods and subsequent diffraction using a traditional cryo-TEM, paving the way for mix-and-spray time-resolved MicroED.

## 5. Related literature

The following references are cited in the supporting information for this article: Bankapalli *et al.* (2020 ▸), Haas *et al.* (2022 ▸), Smith & Wilson (2017 ▸), Thornalley (2003 ▸) and Vander Jagt (1993 ▸).

## Supplementary Material

PDB reference: proteinase K, spray-frozen, 9ozu

PDB reference: Vitrobot-frozen, 9oyy

PDB reference: Vitrobot-frozen with ashless blotting, 9zbf

Extension of spray-freezing methods to Hsp31 microcrystals, and supplementary figures and tables. DOI: 10.1107/S2052252526005129/car5006sup1.pdf

## Figures and Tables

**Table 1:** Statistics of crystallographic data reduction and refinement for proteinase K

	Proteinase K (Vitrobot-frozen)	Proteinase K (spray-frozen)	Proteinase K (Vitrobot-frozen with ashless blotting)
Data collection and processing			
PDB code	9oyy	9ozu	9zbf
No. of crystals merged	3	7	3
Resolution (Å)	32.94–2.20 (2.30–2.20)	48.08–2.50 (2.60–2.50)	48.73–2.20 (2.30–2.20)
Space group	*P*2_1_	*P*2_1_	*P*2_1_
*a*, *b*, *c* (Å)	38.30, 129.10, 47.70	38.33, 128.66, 48.04	39.01, 131.18, 48.73
α, β, γ (°)	90, 90.90, 90	90, 90.52, 90	90, 90.41, 90
Total No. of reflections	120010 (14160)	118430 (6452)	120314 (14183)
No. of unique reflections	36443 (4547)	13435 (1344)	19050 (2392)
*R*_merge_ (%)	26.6 (58.2)	36.3 (113.6)	26.1 (68.7)
CC_1/2_ (%)	90.9 (52.5)	94.0 (49.8)	96.0 (74.2)
〈*I*/σ(*I*)〉	4.86 (2.42)	2.67 (1.14)	5.18 (2.30)
Completeness (%)	79.1 (79.1)	83.4 (76.3)	76.7 (68.7)
Phasing			
Search-model PDB code	2id8	2id8	2id8
Refinement			
Resolution (Å)	32.94–2.20 (2.30–2.20)	48.04–2.50 (2.60–2.50)	48.73– 2.20 (2.30–2.20)
*R*_work_ (%)	21.67	22.12	25.94
*R*_free_ (%)	24.056	26.06	29.05
No. of protein atoms	4099	4103	4092
No. of ligand/ion atoms	4	0	1
No. of solvent atoms	11	7	4
Average *B* factor (Å^2^)	25.79	40.72	43.81
Ramachandran statistics (%)			
Favored	96.93	97.11	96.39
Allowed	3.07	2.89	3.61
Outliers	0.00	0.00	0.00
R.m.s. deviations			
Bond lengths (Å)	0.009	0.009	0.008
Angles (°)	1.878	2.12	1.55
Clashscore	4.73	9.95	8.77

## Data Availability

All structures are available free of charge under PDB codes 9ozu (spray-frozen microcrystals), 9oyy (traditional plunge-frozen microcrystals) and 9zbf (plunge-frozen microcrystals prepared with ashless blotting paper). All raw diffraction data are available from Zenodo at https://doi.org/10.5281/zenodo.17685113.
