# Supplementary material for: Towards time-resolved MicroED grid preparation using mix-and-inject gas dynamic virtual nozzles
Source: IUCrJ. 2026 Jun 23;13(Pt 4):514–21. doi: 10.1107/S2052252526005129 (PMC13324615; doi:10.1107/S2052252526005129)
Supplement: Supplementary file 1 [file m-13-00514-sup1.pdf]

IUCrJ

Volume 13 (2026)

Supporting information for article:

Towards time-resolved MicroED grid preparation using mix-and-inject gas dynamic virtual nozzles

Jacob A. Summers, Niko W. Vlahakis, Kara A. Zielinski, Sarah Uttormark, Scout Fronhofer, Cole Dolamore, Mark A. Wilson, Lois Pollack, Jose A. Rodriguez, Peter D. Dahlberg and Soichi Wakatsuki

S1. Extension of spray-freezing methods to Hsp31 microcrystals

After successfully solving spray-frozen structures of proteinase K, we attempted spray-freezing with crystals of a larger molecular weight protein. For this, we used *Candida albicans* heat-shock protein 31 (Hsp31/Glx3), a member of the DJ-1 superfamily with glyoxalase and chaperone activities (Hasim *et al.*, 2014). As a glyoxalase, Hsp31 catalyzes the conversion of electrophilic natural product methylglyoxal to lactate (Hasim *et al.*, 2014). Methylglyoxal is produced through the spontaneous dephosphorylation of glyceraldehyde-3-phosphate and dihydroxyacetone phosphate and is therefore an unavoidable side-product of central carbon metabolism (Haas *et al.*, 2022). Because methylglyoxal can adduct to multiple cellular nucleophiles to form varied glycation products, its detoxification is an important cellular maintenance task typically performed by the glyoxalase I/II system (Thornalley, 2003; Vander Jagt, 1993). Unlike the better-characterized glyoxalase I/II system, DJ-1 superfamily glyoxalases do not require glutathione as a co-substrate (Bankapalli *et al.*, 2020). Because multiple clades of the DJ-1 superfamily possess glyoxalase activity but have different active sites and catalytic proficiencies (Smith & Wilson, 2017), the structural basis of this enzymatic activity is an intriguing open question and could eventually be addressed by time-resolved MicroED. To this end, we performed spray-freezing with Hsp31 and successfully deposited crystals to grids. However, the deposition rate of crystals on the grids was less than 1 diffracting crystal per 8 grids for Hsp31, making data collection prohibitively difficult. As a consequence, we were only able to compare diffraction patterns obtained at low tilt angles. Despite these limitations, a comparison of traditionally-frozen crystals to spray-frozen crystals demonstrates that the diffraction quality of the Hsp31 crystals was comparable between the two freezing methods (Fig. S4). Despite our ability to measure diffraction from some spray-frozen crystals of Hsp31, we struggled to obtain a dataset with sufficient completeness to determine a high-quality structure of Hsp31 from spray-frozen crystals.

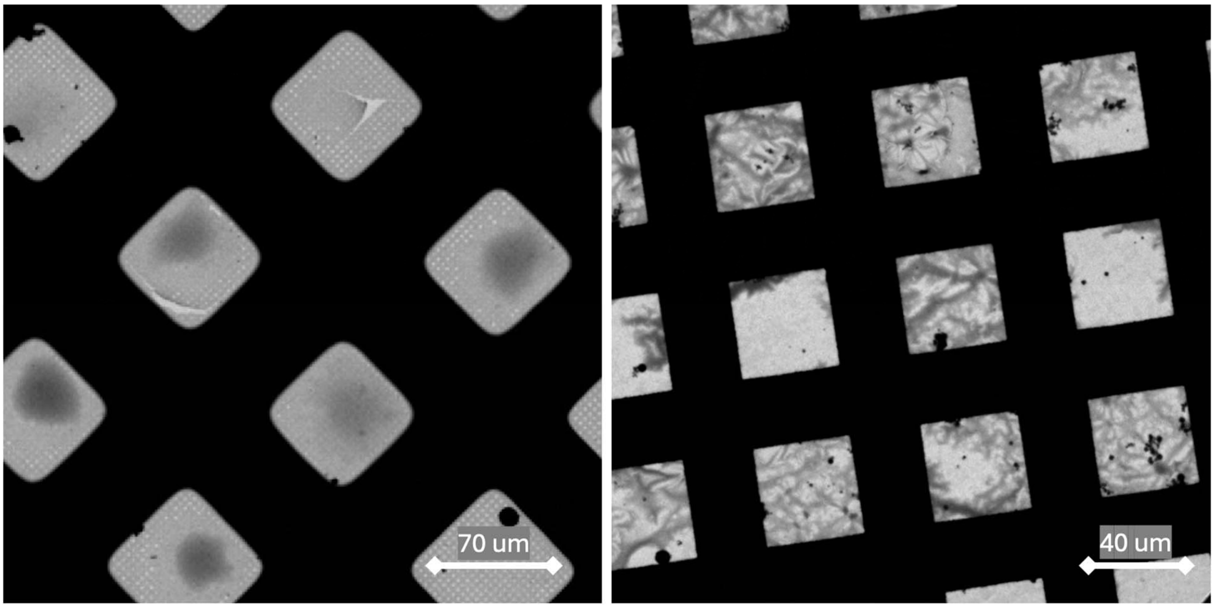

Figure S1 Comparison of ice distribution between spray-frozen crystal solution on 300-mesh Quantifoil (left) compared to 400-mesh lacey carbon (right). On areas of the grid which contained droplets, ice distribution was more variable and thinner sheets of ice were consistently identified using 400-mesh lacey carbon. Comparatively, ice tended to settle in the center of Quantifoil grids without wicking as well as lacey carbon grids.

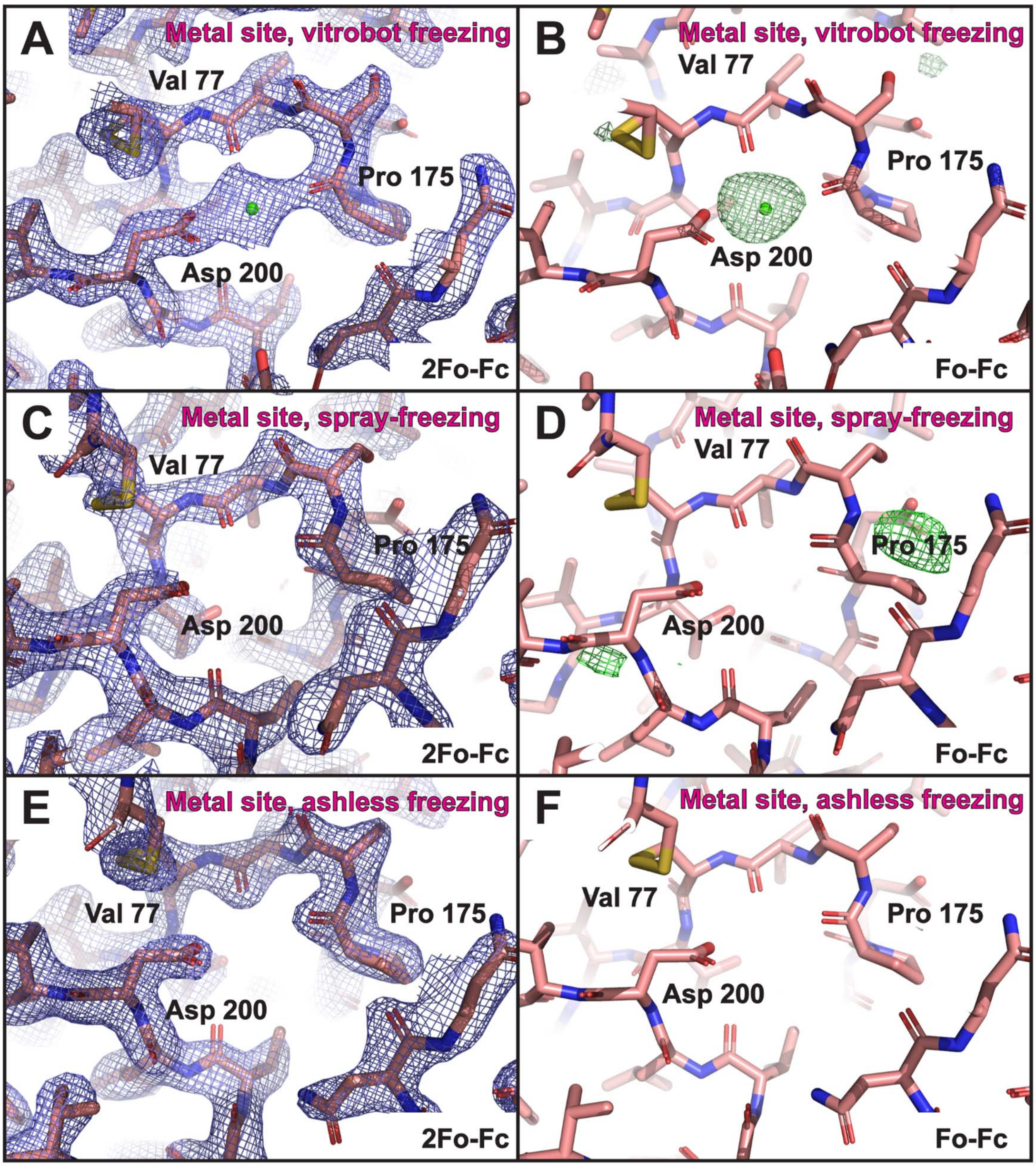

Figure S2 View of the calcium binding site neighboring residue Asp200 from a 2.2 Å resolution structure of proteinase K from crystals prepared by Vitrobot-blotting and plunge freezing methods standard for CryoEM superimposed with the 2Fo-Fc map at 1.5 σ levels (A), and the same calcium site with Fo-Fc map calculated omitting this ion from the model at 3 σ levels (B). The same views are shown for the 2.5 Å resolution structure determined from spray frozen crystals (C-D), and a 2.2 Å resolution structure determined from Vitrobot-frozen crystals prepared with ashless blotting paper (E-F), where in each case a calcium ion is not detected.

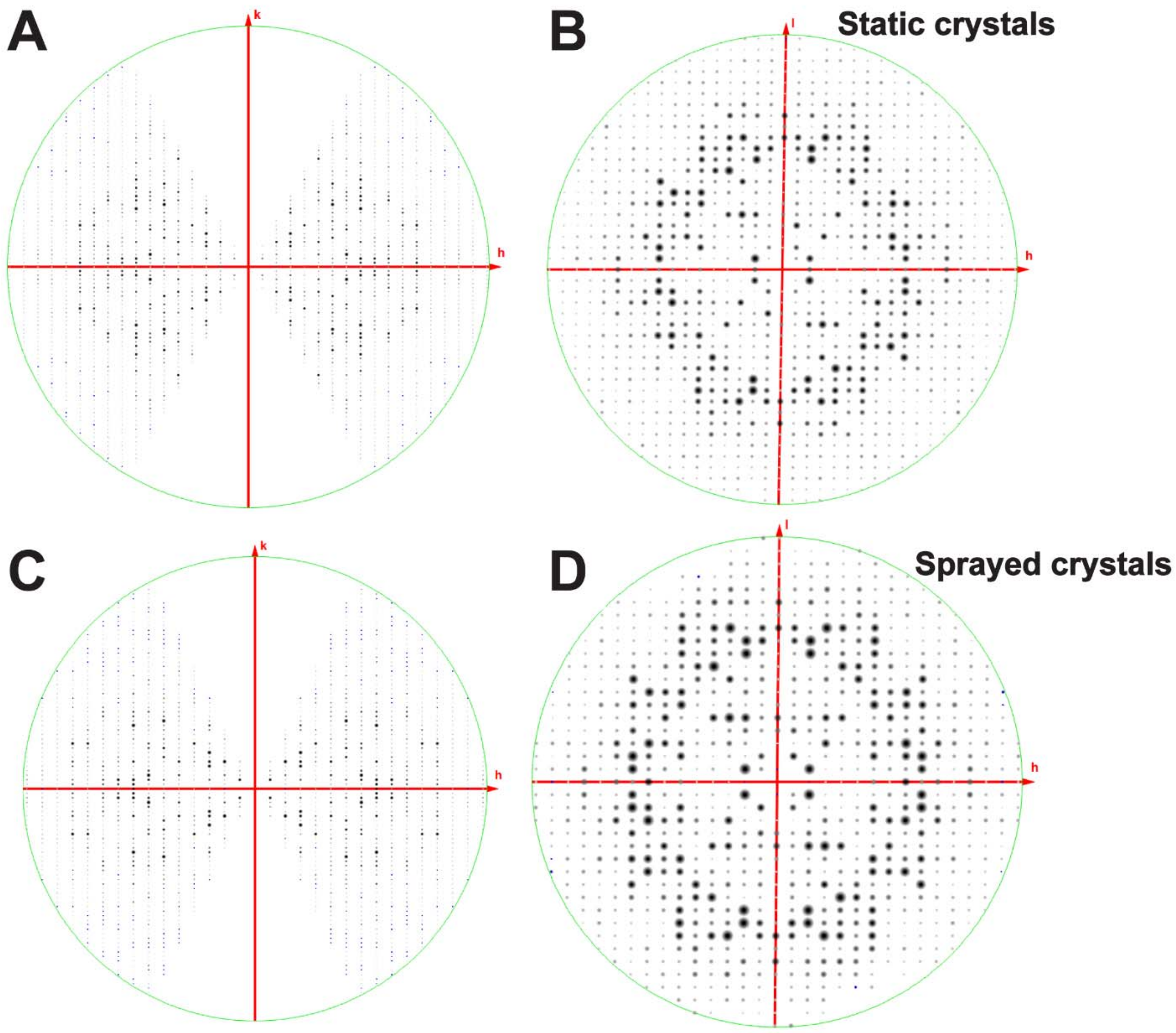

Figure S3 Diffraction angle spot graph showing missing wedges of data acquired from spray-frozen proteinase K microcrystals due to their preferential orientations with b* axis nearly always perpendicular to the grids. Graphic representation from ViewHKL of measured reflections on zone planes (l=0: panels A and C showing missing wedge of about +/- 25 to 30 degrees; k=0: panels B and D) in merged MicroED datasets acquired from three Vitrobot-frozen proteinase K crystals (A & B) and six spray-frozen crystal (C & D). Graphics were generated from lists of scaled reflections, and the relative intensities displayed are self-consistent within panels but not comparable to those in other panels of the figure.

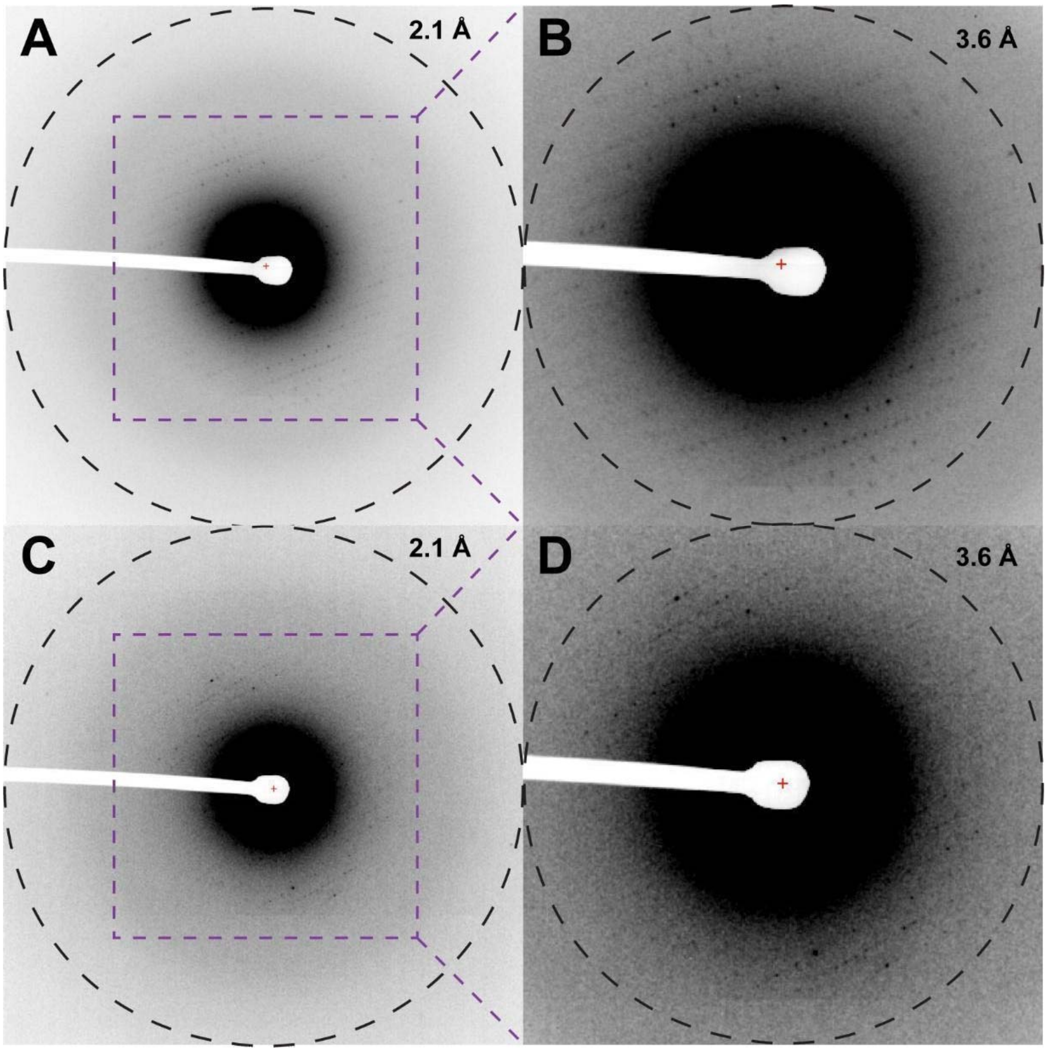

Figure S4 Diffraction comparison between spray-frozen (A-B) and Vitrobot-frozen (C-D) Hsp31 microcrystals show comparable diffraction patterns. Panels B and D show magnified views of the regions of the detector boxed in A and C, respectively, with image contrast adjusted to aid in viewing of low-resolution reflections. Identifying crystals in thin ice remains a difficult challenge, but clear spots can be identified in both diffraction series.

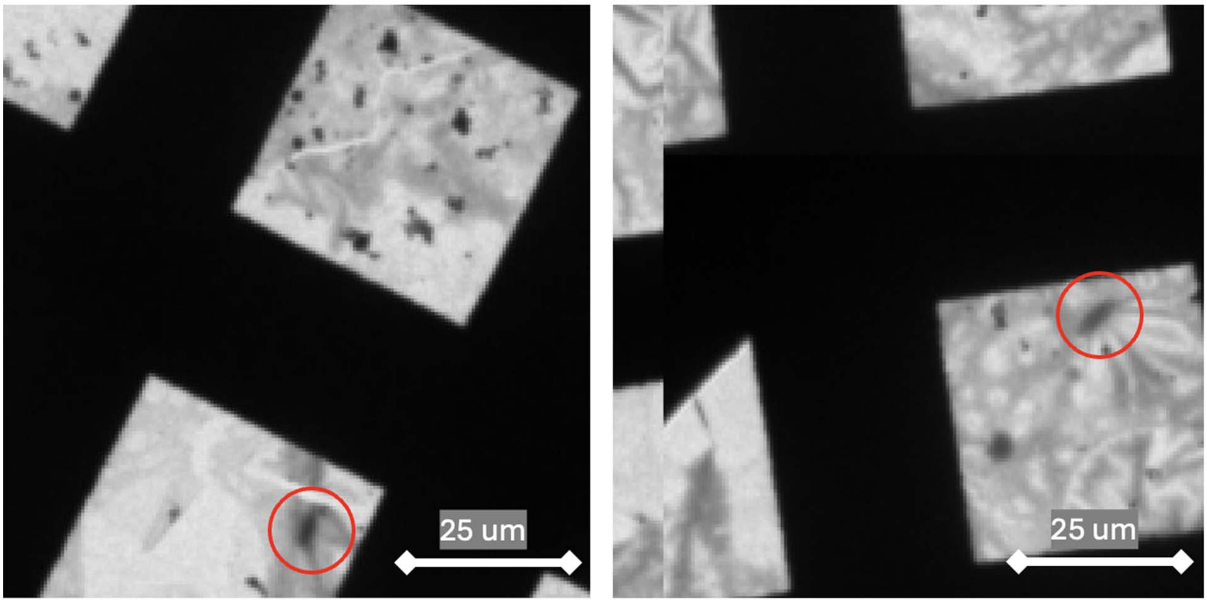

Figure S5 Hsp31 microcrystals spray-frozen onto 400-mesh lacey carbon grids. Differentiating protein crystals from non-vitreous ice and identifying diffracting crystals in thin ice remains difficult.

Table S1 Data Collection and processing statistics of crystallographic data reduction from individual vitrobot-frozen Proteinase K crystals used for merging and structure determination (PDB ID 9OYY)

	Crystal 1	Crystal 2	Crystal 3
Resolution (Å)	32.9 – 2.20 (2.30 – 2.20)	29.8 – 2.20 (2.30 – 2.20)	30.1 – 2.20 (2.30 – 2.20)
Space Group	$P2_1$	$P2_1$	$P2_1$
a, b, c (Å)	38.31, 128.17, 47.70	38.55, 127.32, 47.70	38.46, 128.32, 47.44
α, β, γ (°)	90, 90.32, 90	90, 90.90, 90	90, 90.68, 90
# total reflections	36917 (4228)	42035 (4992)	41089 (4923)
# unique reflections	25434 (3032)	30415 (3758)	23534 (2959)
Rmerge (%)	20.9 (36.0)	8.6 (28.7)	19.7 (61.2)
CC1/2 (%)	84.4 (54.6)	98.2 (68.5)	92.8 (41.7)
<I/σI>	2.12 (1.19)	5.27 (2.52)	2.99 (1.29)
Completeness (%)	55.0 (52.6)	66.0 (65.3)	51.1 (51.5)

Table S2 Data Collection and processing statistics of crystallographic data reduction from individual spray-frozen Proteinase K crystals used for merging and structure determination (PDB ID 9OZU)

	Crystal 1	Crystal 2	Crystal 3	Crystal 4	Crystal 5	Crystal 6	Crystal 7
Resolution (Å)	20.27 – 2.50 (2.60-2.50)	38.70 – 2.50 (2.60-2.50)	44.89 – 2.80 (2.90-2.80)	28.55 – 2.80 (2.90-2.80)	30.07 – 2.80 (2.90-2.80)	32.97 – 2.50 (2.60-2.50)	29.86 – 2.50 (2.60-2.50)
Space Group	$P2_1$	$P2_1$	$P2_1$	$P2_1$	$P2_1$	$P2_1$	$P2_1$
a, *b*, *c* (Å)	38.33, 128.72, 48.01	38.70, 129.95, 47.99	38.72, 127.14, 47.97	38.37, 128.23, 48.10	38.47, 129.76, 48.02	38.34, 129.23, 48.24	38.56, 127.59, 48.01
α, *β*, *γ* (°)	90, 90.47, 90	90, 90.20, 90	90, 90.62, 90	90, 90.76, 90	90, 90.16, 90	90, 90.04, 90	90, 90.81, 90
# total reflections	15819 (1140)	8771 (378)	11003 (949)	13440 (1105)	10031 (702)	18382 (1555)	23189 (1738)
# unique reflections	7344 (635)	4423 (298)	5726 (525)	5537 (484)	3682 (291)	6076 (567)	9075 (824)
R_{merge} (%)	11.9 (35.1)	9.8 (44.9)	16.3 (85.4)	21.2 (76.3)	15.2 (48.2)	20.4 (119.8)	15.7 (65.1)
CC1/2 (%)	97.2 (80.4)	97.6 (78.4)	96.1 (14.4)	94.3 (42.0)	96.5 (65.0)	96.7 (27.9)	98.2 (52.6)
<I/σI>	5.44 (2.00)	6.16 (1.25)	4.12 (0.808)	3.37 (1.04)	6.28 (2.31)	4.98 (0.94)	6.02 (1.29)
Completeness (%)	45.6 (36.0)	26.9 (16.1)	50.4 (47.5)	48.3 (42.5)	31.7 (25.6)	37.3 (31.2)	56.4 (46.1)

Table S3 Data Collection and processing statistics of crystallographic data reduction from individual vitrobot-frozen Proteinase K crystals treated with ashless blot paper used for merging and structure determination (PDB ID 9ZBF)

	Crystal 1	Crystal 2	Crystal 3
Resolution (Å)	45.68 – 2.20 (2.30-2.20)	48.58 – 2.20 (2.30-2.20)	48.65 – 2.20 (2.30-2.20)
Space Group	$P2_1$	$P2_1$	$P2_1$
a, *b*, *c* (Å)	39.01, 131.18, 48.73	39.06, 131.80, 48.58	39.08, 132.55, 48.65
α, *β*, *γ* (°)	90, 90.41, 90	90, 90.70, 90	90, 90.39, 90
# total reflections	39436 (4794)	40086 (4891)	40962 (4547)
# unique reflections	13813 (1710)	13288 (1665)	14302 (1717)
R_{merge} (%)	14.9 (40.4)	16.1 (42.2)	22.8 (97.6)
CC1/2 (%)	97.8 (77.6)	97.6 (83.5)	95.5 (54.2)
<I/σI>	4.82 (2.15)	4.58 (2.17)	3.01 (0.91)
Completeness (%)	55.6 (55.0)	53.3 (53.2)	57.0 (54.5)
